# A Diverse and Flexible Teaching Toolkit Facilitates the Human Capacity for Cumulative Culture

**DOI:** 10.1007/s13164-017-0345-4

**Published:** 2017-06-01

**Authors:** Emily R. R. Burdett, Lewis G. Dean, Samuel Ronfard

**Affiliations:** 1 School of Psychology and Neuroscience, University of St. Andrews, St Andrews, Fife, KY16 9JP UK; 2 Graduate School of Education, Harvard University, Boston, MA USA

## Abstract

Human culture is uniquely complex compared to other species. This complexity stems from the accumulation of culture over time through high- and low-fidelity transmission and innovation. One possible reason for why humans retain and create culture, is our ability to modulate teaching strategies in order to foster learning and innovation. We argue that teaching is more diverse, flexible, and complex in humans than in other species. This particular characteristic of human teaching rather than teaching itself is one of the reasons for human’s incredible capacity for cumulative culture. That is, humans unlike other species can signal to learners whether the information they are teaching can or cannot be modified. As a result teaching in humans can be used to support high or low fidelity transmission, innovation, and ultimately, cumulative culture.

## Introduction

Although some authors argue that culture is not a uniquely human adaptation, human culture is uniquely complex (Dean et al. 2014; Laland and Galef, 2009). In particular human culture is cumulative – that is, it develops and becomes more and more complex by building on the knowledge and cultures of previous individuals and groups over generations (Dean et al. 2014; Tennie et al. 2009; Boyd and Richerson 1996). Over time cumulative culture results in cultural traits (e.g. behavior patterns, artefacts, traditions) that are more complex than one individual could have invented alone (Tennie et al. 2009).

As culture becomes increasingly complex, these cultural traits are less likely to be learned solely by inadvertent social learning mechanisms (henceforth ‘social learning’). For example, building a helicopter or performing open-heart surgery requires a high standard of acquired knowledge as well as a certain level of experience before one could consistently be successful at either pursuit. Thus, high-fidelity copying is important for the occurrence and persistence of cumulative culture (Tomasello 1999; Lewis and Laland 2012; Galef 1992; Boyd and Richerson 1996). Without high-fidelity transmission of cultural traits, each individual would be required to reinvent large parts of those traits (Tennie et al. 2009). The costs of time and effort required to learn cultural traits from low-fidelity transmission therefore impose an upper limit on what cultural knowledge can be transmitted. Imitation and teaching are examples of high-fidelity transmission mechanisms. Whereas low-fidelity transmission allows for the adaptation and potential innovation of skills and knowledge, high-fidelity transmission reduces the loss of information during transmission, thus reducing the time and energy costs of learning (Lewis and Laland 2012; Boyd and Richerson 1996) and increases the likelihood of successful transmission.

The importance of teaching to the persistence of human cumulative culture is that it is a high- fidelity mechanism that allows cultural traits to be accurately passed on (Tomasello 1999, Galef 1992 although see Claidière et al. 2014; Zwirner and Thornton 2015 for alternative views, Dean et al. 2014). High-fidelity transmission must, however, be balanced with the ability to alter cultural traits, if cumulative cultural evolution is to occur. Therefore, a twin driver of cumulative cultural evolution is innovation (Legare and Nielsen 2015; Enquist and Ghirlanda 2007). Innovative changes can occur through a range of mechanisms for example, through combinations of cultural traits or modifications to a single trait (Lewis and Laland 2012; Slater and Lachlan 2003), building upon the efforts of previous generations. Changes may be intentional and insightful, or the result of errors and accident. For example, knowledge has spread, has been modified, and has been transmitted and accumulated in fields as diverse as technology (Basalla 1989), mathematics (Mesoudi 2011), and symphonic music (Lehman 1947).

Many researchers have argued that humans are not the only species to demonstrate the capacity to create culture. Indeed, chimpanzees (Whiten et al. 1999) and crows (Corvus) (Hunt and Gray 2003) are well-known for their complex traditions and socially-learned skills. Although there is considerable debate about whether other species exhibit teaching, there is no question that human teaching is more diverse, flexible, and complex in humans than in other species. This uniqueness of human teaching, then, rather than teaching itself may be the key to the accurate transmission and spread of knowledge and to the evolution of culture.

In this article, we put forth the argument that our species’ diverse teaching toolkit allows us to modulate the normative strength of our teaching. This ability to signal through diverse teaching strategies and verbal clarifications whether a learner should engage in high-fidelity copying or whether there is flexibility to modify and innovate, allows humans to balance learning, teaching, and innovation, affecting cultural evolution across behavioral domains.

We first argue that teaching is one of the key drivers of the process of cumulative culture because it provides a system of imparting knowledge that has become too complex to learn through social learning. We review research that shows that although human and some non-human populations demonstrate teaching, instances of spontaneous teaching in open conditions are relatively rare. We discuss reasons for the scarcity of such teaching by discussing the costs of providing and seeking teaching. We then argue that beyond these costs, another reason for the relative rarity of teaching (particularly direct verbal teaching) is that humans use a variety of teaching strategies in a manner that balances the need for high-fidelity and low-fidelity transmission with innovation. Finally, we conclude with directions for future research.

## Teaching is (Relatively) Rare

Teaching was operationally defined by Caro and Hauser (1992) as a teacher modifying their behavior to facilitate learning in a naïve observer with no immediate benefit (or some cost) to the teacher. It is rare across the animal kingdom and shows an unusual phylogenetic spread (Fogarty et al. 2011; Hoppitt et al. 2008). For example, there is evidence of teaching in meerkats and ants, but little evidence in our closest evolutionary cousins, chimpanzees (Hoppitt et al. 2008). This evidence has led to the theory that teaching will only be favored by selection where the costs for individuals to teach are outweighed by the long-term fitness benefits and other learning options are not available (Fogarty et al. 2011; Thornton and Raihani 2008). We deliberately employ a functional definition of teaching in this paper, rather than specifying a mechanism by which teaching may occur across species. While the unusual phylogenetic spread and different forms it takes across species suggests teaching is not a single biological function, the focus of this paper is the function and evolution of teaching, not its mechanism (Tinbergen 1963).

Meerkats (*Suricata suricatta*) offer a case-study. Juvenile meerkats are taught to hunt scorpions by adults in the group (Thornton and McAuliffe 2006). Scorpions present a nutritious foodstuff for meerkats, but the sting can kill a meerkat if the scorpion is not processed properly. Adults therefore bring younger pups dead scorpions to eat, and gradually expose older pups to scorpions from which they have removed the sting and subsequently supervise pups killing a scorpion with a sting. Killing a scorpion would be difficult and dangerous to learn by trial-and-error learning or by social learning, both of which may result in death of the pup. Therefore, the cost of teaching for the individual meerkats, are outweighed by the kin-selection benefits. If more juveniles can utilize the nutritious scorpions as food, then the population is likely to grow.

This case study illustrates the form of teaching in non-human animals. Where it has been found, teaching solves complex problems in a specific domain; hunting in meerkats (Thornton and McAuliffe 2006), fledging in pied babblers (Raihani and Ridley 2008), and navigation in ants (Franks and Richardson 2006). In contrast, teaching in humans is characterized by its flexibility and domain-generality (Hoppitt et al. 2008; Kline 2015; Caro and Hauser 1992). Human teaching is so broad that in some populations it now includes school curricula.

Anthropologists, however, have highlighted that while modern Western populations have formalized school systems and ‘helicopter parenting’, teaching may be rarer or look different in other populations around the world (Hewlett and Roulette 2016; Kline 2015). Some anthropologists have even questioned whether teaching is a human universal (Lancy 2010; Paradise and Rogoff 2009). These researchers have highlighted the importance of forms of inadvertent social learning for children, with children copying those who appear to be more expert at tasks. This inadvertent social learning, however, may be shaped by adults or older peers, giving children the opportunity to learn new skills or knowledge. This shaping of children’s opportunities and exposure to new stimuli has recently been highlighted as a form of teaching (Kline 2015; Hewlett and Roulette 2016). This work suggests that teaching is likely to be a human universal and human teaching strategies may form a continuum that range from explicit to more implicit – a point we return to below.

Young children engage in explicit teaching and frequently do so when prompted. When asked to teach a naïve peer, young children will demonstrate and explain how to play a game, complete a task, or how to use an object (Ashley and Tomasello 1998; Davis-Unger and Carlson 2008a, b; Davis-Unger and Carlson 2008a, b; Ronfard and Corriveau 2016; Strauss and Ziv 2012; Strauss et al. 2002; Wood et al. 1995). The ability of children to engage in explicit teaching develops throughout preschool. Three-and four-year-old children mostly demonstrate while five-year-olds explain (e.g., Strauss et al. 2002). Moreover, with age, children not only become more explicit teachers who directly address their pupil’s mistakes they also become better at tailoring the amount of information they provide in response to mistakes (Ronfard and Corriveau 2016). However, where it has been tested in an ‘open’ condition (i.e. children have not been instructed to teach, but the experimental set-up allowed participants to teach when they chose to), some but not frequent teaching occurs. For example, Dean et al. (2012) explored whether cumulative culture could occur in an experiment using a puzzle box with three increasingly difficult ‘levels’, with each level giving a better reward. In contrast to capuchins and chimpanzees, children were found to teach. It should be noted, however, that in 35 children, all exposed to the puzzle box for 150 mins only 23 instances of teaching were recorded. It appears that the influence of social learning and individual trial-and-error learning was greater than the impact of teaching on individual learners.

These examples highlight that at each level, across non-human and within human species, within populations or within small groups, spontaneous teaching is relatively rare. It seems that a large amount of information can be transmitted via low-fidelity social learning or learned through trial-and-error.

## The Cost of Teaching in Humans

One reason that teaching is relatively rare in open conditions is that teaching is costly. It requires an investment of time and resources (Fogarty et al. 2011; Thornton and Raihani 2008; Caro and Hauser 1992; Kline et al. 2013). This is true for teachers and learners. That is, there is a *cost of providing teaching* and there is a cost of *requesting teaching*. For example, *for the learner*, receiving good teaching can be one of the most optimal and efficient ways to gain expertise but gaining access to an expert may be difficult or depend on the resources of the learner to persuade an expert to invest (particularly if the teacher is not related to the learner). In addition, requesting teaching is costly because it opens one to being misled. Information is valuable and teachers may not want to share that information with anyone. In fact, in pre-contact Hawaii, deep sea fishermen only shared the location of productive fishing grounds with close family members (Kamakau 1976). Similarly, in Maine’s Middle Harbor, lobstermen were secretive about fruitful fishing locations, sharing information selectively with kin and the equally skilled (Palmer 1991). *For the teacher*, providing instructions can be a way to gain prestige or to acquire resources, but teaching takes time and commitment. Thus, long term or extensive teaching which is necessary for learning complex and opaque skills likely requires some form of reciprocity and cooperation between the teacher and the learner in order for both sides to feel they have been given or have received value. There are several ways for the learner and teacher to “balance the scales”.

One way for teachers and learners to “balance the scales” is to restrict teaching to established close relationships. In these relationships there is a higher likelihood that these individuals share similar social status, family connections, skill, or age and thus a higher likelihood of an established reciprocation of teaching and other altruistic acts. Thus individuals may continue to teach others with the expectation that the act will be reciprocated further in the future (Trivers 1971). By teaching key knowledge and skills that increase survival to close kin and offspring, an individual can increase their inclusive fitness (Cavalli-Sforza and Feldman 1981; Castro and Toro 2014). At a proximate level, a learner is more likely to have access to close-kin models as teachers making these more likely teachers (Henrich and Broesch 2011). Beyond one’s family, one’s clan (or cultural group) is also likely to have been an important source of support. Particularly if one’s cultural group had developed adaptive cultural traits that encouraged reciprocity and the enforcement of cultural norms (see (Boyd and Richerson 1985). Indeed, children are particularly likely to correct and reteach norms to others when individuals are part of their in-group rather than outgroup (Schmidt et al. 2012). In sum, teaching within close kin or cultural relationships is cheaper for the learner, and there are likely to be direct and indirect benefits for the teacher.

Another way for teachers and learners to reduce their respective costs and increase the value of the instruction for both sides is through a system of balanced power. Learning from an expert does not always guarantee that the learner will acquire the knowledge or skill he will need to acquire improvement. This can be because not all experts have extra amounts of time to spend teaching learners and some experts are not the best teachers. If teaching takes valuable time away from an expert, a more strategic way to learn a skill is for the learner to seek out “lesser experts”, or those individuals who are 1 or 2 steps above the learner (Henrich 2015). These individuals may have a better grasp of the units of improvement needed for the learner to achieve the next level of learning compared to an expert who might not remember or be able to communicate adequately the necessary steps to achieve a learning goal. A further benefit is that similar aged peers are likely to be or become friends, thus there may be more incentive to help each other on new tasks. Thus, a learner receives teaching at an appropriate level, the “junior” teacher advances in respect and prestige, and the relationship has the potential to increase social bonds and friendship. Related work using simple tasks suggests that preschool children prefer to learn from adults (Wood et al. 2012; McGuigan et al. 2011) and familiar caregivers (Lucas et al. in press; Harris and Corriveau 2011; Corriveau and Harris 2009; Corriveau et al. 2009). But other studies have shown that children also prefer to learn from same-aged peers in a particular context (i.e., when an object is seen as a toy) (Wood et al. in press; VanderBorght and Jaswal 2009) and older children (> age 6) are more likely to learn from (unfamiliar) experts (Lucas et al. in press). However, a more difficult task (requiring learning a complex skill) may reveal that children seek out more junior experts and teachers. And, although no study has directly looked at social bonding between teacher and learner, other work suggests that imitating or doing something in synchrony increases liking and affiliation (Tuncgenc and Cohen 2016; Over and Carpenter 2013; Over et al. 2013; Over and Carpenter 2012).

A third way for instruction to be maximally effective is to restrict teaching to hard-to-learn rather than easy-to-learn information. That is, because teaching takes time and effort teachers have an incentive to distinguish between information that the learner can learn on her own and information the learner cannot learn alone and to focus instruction on the former. Indeed, anthropological and experimental work suggest that harder to acquire skills are more often taught than easier to acquire skills (Kline et al. 2013; Ronfard et al. 2016). Other empirical work suggests that learners benefit more from verbal teaching when learning a complex skill (stone knapping)—compared to simple imitation or learning from a non-verbal teacher who uses gestures)—as demonstrated by their ability to produce better quality tools across several generations (Morgan et al. 2015). Thus, direct verbal teaching is particularly beneficial when high-fidelity transmission is necessary for learning a complex skill or a cultural norm. In other learning situations, when a skill or object function is not overly complex or could benefit from adaptation to a new circumstance, lower-fidelity transmission mechanisms like imitation or less explicit teaching may be preferable.

In sum, the rarity of spontaneous teaching in “open” paradigms is not surprising. Teaching is costly for teachers and for learners and as a result, occurs more frequently with kin and in-group members rather than non-kin and out-group members. It also occurs more frequently for harder rather than easier-to-learn information (Kline et al. 2013; Ronfard et al. 2016; Morgan et al. 2015).

## How Does Human Teaching Balance Transmission Fidelity and Innovation?

The fact that spontaneous teaching in “open paradigms” is relatively rare may actually be a clue as to why the development of cumulative cultural traits is so prevalent in humans. As we discussed in the introduction, teaching is hypothesized to support cumulative culture by supporting the high fidelity transmission of information. However, cumulative culture not only requires high fidelity transmission, it also requires that cultural traits are modified and adapted by innovations through low-fidelity transmission (Legare and Nielsen 2015) that are themselves transmitted over time. A challenge for the establishment of cumulative culture is to balance the conservatism produced by teaching and other high-fidelity mechanisms with the flexibility necessary for innovation. We argue that human teaching is able to balance the need for higher and lower transmission fidelity because human teachers have at their disposal a diverse teaching toolkit. Human teachers’ selection of a particular teaching strategy from their diverse teaching toolkit allows them to signal to learners when higher fidelity is required and when lower fidelity (and deviation) is appropriate.

Before unpacking this argument, it is worth quickly returning to a point we made in the previous section. Teachers have an incentive to restrict their teaching to knowledge and skills that are difficult for a learner to acquire on her own. This selection of what to teach is itself a means for teachers to balance the need for high transmission fidelity and flexibility. By not explicitly teaching some skills and letting learners figure them out on their own, teachers introduce some flexibility in the development of these less complex skills. Thus, by allowing or discouraging high-fidelity learning, teachers can provide a fertile ground for innovation borne out of random errors or deliberate experimentation.

However, if teachers decide that teaching is needed (i.e., because the learner cannot acquire the information on her own), how might they signal to learners whether the information they are teaching can or cannot be modified? Kline (2015) has proposed a taxonomy of teaching behaviors that range from the less to the more explicit: teaching by social tolerance (i.e., allowing the learner to observe closely even if such observation is intrusive), teaching by opportunity provisioning (i.e., creating opportunities for the learner to practice), teaching by stimulus enhancement (i.e., guiding the attention of the learner to relevant aspects of the task), teaching by evaluative feedback, and direct active teaching (i.e. giving instruction to the learner about how to complete a task, tailored to the learners experience and needs). Humans are the only species to engage in all five forms of teaching and they are the only species to engage in direct active teaching. This is noteworthy because human’s ability to use multiple teaching strategies as well as the ability to provide direct verbal instructions allows teachers to influence the degree of fidelity and flexibility that their teaching generates.

Using Kline’s (2015) framework to think about the diversity and flexibility of the human teaching toolkit provides a possible explanation for relative rarity of teaching in “open paradigms”. The type of teaching that has been coded in these experiments is direct teaching. This is noteworthy because direct teaching may be particularly likely to generate high fidelity transmission. This is because humans appear to process information differently when they realize that it is being produced for their benefit (Csibra and Gergely 2009). Specifically, human infants appear ready to generalize information that is conveyed to them pedagogically. Modelling and experimental work suggests that learners make stronger inferences when they are directly taught because this reduces the number of hypotheses they consider and this allows them to make stronger inferences from less information (Shafto et al. 2014; Shafto et al. 2012). In support of this argument, Bonawitz et al. (2011) find that children who are taught about a novel toy subsequently restrict their exploration of that toy to the demonstrated function (explore less) and thus discover fewer functions of the toy than children who were not taught about the toy. This strand of research implies that direct verbal teaching may reduce innovation by lessening independent exploration and thus the chance to discover novel information. However, most of the teaching that humans, both across and within cultures, engage in does not fall into the category of direct active teaching. Moreover, it is not clear that other types of teaching (for example, opportunity provisioning) would generate similarly strong inferences and higher fidelity imitation on the part of learners that would reduce exploration and innovation. This is because in the case of opportunity provisioning the teacher is not directly providing information for the learner and is instead giving the learner opportunities to learn on her own that the learner would not otherwise have had. Thus, being taught opportunity provisioning may not lead learners to feel beholden to a particular method of completing a task – a claim that could easily be tested.

In addition to allowing the learner to make stronger inferences, direct active teaching and particularly verbal teaching allows teachers to directly control whether children will perceive the information they are taught as needing to be reproduced with fidelity rather than with some degree of flexibility. The default impact of direct verbal teaching may be to generate the highest level of imitative fidelity and the lowest potential for experimentation on the part of the learner. However, teachers can moderate this default effect by clarifying their intent through language. For example, the fidelity of children’s reproduction of causally unnecessary actions embedded within a task they are taught to complete varies depending on how the task is framed by the teacher. Children are more likely to faithfully imitate and teach other people inefficient actions when these actions were presented using a conventional framing rather than an instrumental one (i.e., “this is how we do it” vs. “this is how she always does it”) (Clegg and Legare 2016).

Likewise, children may be less likely to faithfully imitate when children are not given direct or normative language and encouraged to explore. For example Kittredge et al. (2014) examined whether the negative effect of instruction on exploration reported by Bonawitz et al. (2011) could be remedied by the addition of a simple clarification by the teacher. They introduced children to a diorama of a forest where they had hidden multiple animals. They tasked children with finding as many animals as they could. Some children received no instructions (control). Other children were taught one way to look for animals – “here’s how you can find animals” (instruction). A third group of children were taught how to look for the animals but were also given a hint – “here’s how you can find animals...but there could be lots of other ways to find animals” (instruction + clarification). They replicated the finding of Bonawitz et al. (2011) that children who received instructions focused on the demonstrated strategy, explored less, and found fewer animals than children in the control condition. However, this effect of instruction on exploration disappeared in the instruction + clarification condition. That is, when children were told that there may be other ways to find animals, they used the demonstrated strategy but also went beyond it and were equally effective as children who did not receive any instructions.

Kittredge et al. (2014)‘s study makes a powerful point. The ability of human teachers to clarify their pedagogical intent means that even if direct verbal teaching leads learners to engage in high fidelity copying and restricts exploration (and thus perhaps innovation) that effect can be moderated by simply making clear to the learner that alternative strategies are available. By making known there are alternative strategies, children may be more inclined to low fidelity copying and further exploration. This human ability to explicitly state the goal of their pedagogy and the inferences they wish the learner to make from a set of pedagogical cues is likely to be an important reason why humans have built such complex cultures in various parts of the world.

## Future Directions

In this article, we explored how a flexible teaching tool-kit allows humans to deploy teaching as both a mechanism for the high-fidelity transfer of information and as a means to encourage low-fidelity transmission, exploration, and innovation in others. Understanding how teaching develops and how it facilitates cumulative culture is a new and relatively underdeveloped area of research. Below we outline directions for future research.

We have argued that humans have a flexible and diverse toolkit with several strategies for teaching that can be more and less explicit. We further argued that more explicit and direct teaching may hinder innovation because it may be associated with normative implications (i.e., it must be done this way) but that this effect can be reduced by teachers who explicitly state the inferences they want learners to make about the normativity of what they are being taught. This raises additional questions. For example, how do teachers decide when it is appropriate and beneficial for them to teach more or less explicitly? We might predict that complex skills, particular cultural behaviors, or information vital to health and well-being may be more likely to be taught directly, to promote higher fidelity transmission. Additionally, we might expect particular problem solving, engineering skills or particular subjects (e.g., art, creative writing, technology) that prize creativity to be taught using (less explicit) teaching strategies that support the development of elementary skills but encourage innovation (see Gentner et al. 2016 for some initial work on this).

Humans have been teaching one another for generations (Castro and Toro 2014; Tehrani and Riede 2008; Högberg et al. 2015; d'Errico and Banks 2015) thus building up a large corpus of accumulated knowledge. As a result, not all accumulated knowledge by a particular cultural group is taught to every member of the group. Instead, many individuals specialize and attain a particular expertise (Hutchins 1995). Thus, similar questions to the ones just outlined at the individual level can be asked at the group level: how do groups decide what information should be taught, by whom, and in what manner? Investigating how groups developed mechanisms for the transmission and preservation of information and how these decisions where codified and institutionalized is a fertile ground for future research. It is possible that the codification and institutionalization of knowledge transmission (and therefore of the development of specific pedagogical beliefs across various groups and institutions) is a relatively recent phenomena. After all, for specialization to occur and make sense, a group must be large enough to sustain that specialization (Kline and Boyd 2010). But it may be that the development of such codes and institutions was and is an important catalyst for cumulative culture. Codification of the curation of knowledge and of who is involved in that process makes it easier for learners to identify reliable and trustworthy individuals who can provide them with the information they seek. It facilitates horizontal and oblique transmission by rewarding teachers for their labor and thus making it cheaper for learners to seek instruction. And, as institutions form and solidify in their knowledge base, tenure and repute, the information and the leaders that emerge from these venues will be seen as reliable and further generate a virtuous cycle.

In sum, teaching plays an important role in the development of cumulative culture. In its most explicit form, it is a powerful mechanism that ensures high fidelity transmission of information. However, it is not clear that all teaching leads to high fidelity transmission of information. Instead, we have different levels of teaching strategies, particular rules of reciprocity, and institutions that limit or encourage different types of teaching, and in turn, learning and innovation. This diverse and flexible toolkit allows humans the opportunity to modulate high and low fidelity learning by the ability to share knowledge in ways that maintain acquired knowledge and customs and also increase the diversity and evolution of culture.
